# Identification of a novel prognostic gene signature associated with therapeutic resistance in hepatocellular carcinoma

**DOI:** 10.1016/j.gendis.2025.101837

**Published:** 2025-08-30

**Authors:** Siliang Wang, Shuangshuang Wang, Xu Wang, Yuxiang Sun, Xiao Du, Dan Han, Huanyu Ni, Yun Zhu, Huilian Shi, Zhaocong Yang

**Affiliations:** a Department of Pharmacy, Nanjing Drum Tower Hospital, Affiliated Hospital of Medical School, Nanjing University, Nanjing, Jiangsu 210000, China; b Department of Pathology, Jiangsu Province Hospital of Chinese Medicine, Affiliated Hospital of Nanjing University of Chinese Medicine, Nanjing, Jiangsu 210029, China; c Jiangsu Center for the Collaboration and Innovation of Cancer Biotherapy, Cancer Institute, Xuzhou Medical University, Xuzhou, Jiangsu 221000, China; d Institute of Translational Medicine, Medical College, Yangzhou University, Yangzhou, Jiangsu 225009, China; e Department of Infectious Diseases, Jiangsu Province Hospital of Chinese Medicine, Affiliated Hospital of Nanjing University of Chinese Medicine, Nanjing, Jiangsu 210029, China; f Department of Cardiothoracic Surgery, Children's Hospital of Nanjing Medical University, Nanjing, Jiangsu 210008, China

Hepatocellular carcinoma (HCC), globally the fourth-leading cause of cancer death, shows marked heterogeneity.^1^ In China, advanced HCC first-line therapies include sorafenib and oxaliplatin-based chemotherapy to improve overall survival, also used in conversion therapy for unresectable tumors.^2^^,^^3^ However, only 30% of patients respond, with most progressing within six months. Resistance causes adverse effects and significant financial burdens; non-personalized management worsens healthcare inefficiencies.^4^ Current prognostic systems [*e.g.*, Barcelona Clinic Liver Cancer (BCLC) staging, TNM staging] fail to predict therapy responses or survival due to oversimplified criteria,^5^ urgently requiring biomarkers reflecting HCC's molecular complexity.

To address this gap, we developed a gene signature predictive of survival and therapy resistance in advanced HCC. Our findings underscore the importance of biomarker-driven strategies to address HCC heterogeneity, ultimately improving overall survival and reducing the socioeconomic impact of ineffective therapies. This framework may guide future clinical trials exploring personalized neoadjuvant or adjuvant interventions for advanced HCC.

Firstly, we identified therapy-resistance prognostic genes in HCC. Using GEO datasets (GSE62813 for sorafenib and GSE129071 for oxaliplatin), we found 2968 differentially expressed genes in sorafenib-resistant cells and 395 differentially expressed genes in oxaliplatin-resistant cells (*P* < 0.05; Fig. 1A and B; Fig. S1; Tables S3 and S4). The intersection revealed 99 overlapping resistance genes (Fig. S2A). Univariate Cox regression (TCGA-LIHC) linked 27/99 genes to overall survival (*P* < 0.05; Table S5). The consensus clustering using these 27 genes stratified patients into three subtypes (C1–C3), with C2 having the best and C3 worst overall survival (Fig. S2B–S2E). Protein–protein interaction analysis showed connectivity among 22 genes (Fig. S3A). Topological analysis (degree/closeness/betweenness/eigenvector) identified 15 hub genes exceeding median thresholds as key prognostic markers (Fig. S3B). This multistep approach enables precise identification of resistance drivers, facilitating risk stratification and personalized treatment strategies for HCC patients unresponsive to first-line therapies.

**Figure 1:**
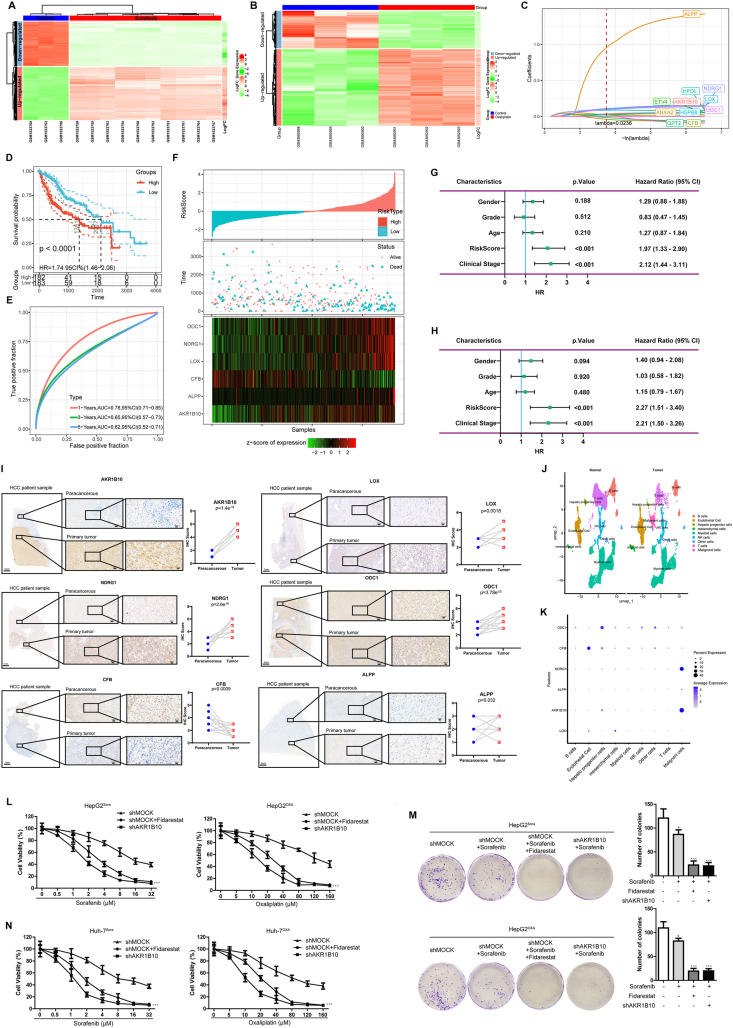
Identification of a novel prognostic gene signature associated with therapeutic resistance in hepatocellular carcinoma (HCC). **(A, B)** Identification and functional annotations of the differentially expressed genes (DEGs) between treatment-sensitive and -resistant HCC cells. (A) The heatmap showing DEGs between HCC cells with sorafenib resistance and sensitivity. (B) The heatmap showing DEGs between HCC cells with oxaliplatin resistance and sensitivity. **(C)** Discovery of a therapeutic resistance-related signature to predict HCC prognosis through LASSO and stepwise regression. Alteration rule of every independent variable. The vertical and horizontal axes stand for the coefficient and log value of the independent variable lambda, respectively. Coefficients were non-zero for the eleven genes at lambda = 0.0236. **(D**–**F)** Performance of our prognosis model constructed based on six therapeutic resistance-related genes in classifying training set samples. (D) Difference in prognosis after classification of training set samples using our six-gene signature. (E) Receiver operating characteristic curves for the six-gene signature for the training set HCC samples. (F) The associations between risk score, survival status, survival time, and six gene expression levels within training set samples. **(G, H)** Clinical independence of the prognosis model constructed by incorporating six genes related to oxaliplatin/sorafenib resistance. Univariate (G) and multivariate (H) Cox regression on risk scores and clinical data of training set samples were carried out for calculating respective hazard ratios (HRs), 95% confidence intervals (CIs), and *P*-values. **(I)** The levels of LOX, ALPP, CFB, ODC1, AKR1B10, and NDRG1 in paracancerous and primary HCC tumor samples were detected by immunohistochemistry (IHC). Right, IHC scores of the proteins. **(J)** UMAP showed the distribution of annotated cell subgroups. **(K)** The bubble diagram showed the expression of six prognostic genes in the cell subgroups we annotated. The more purple the color, the higher the average expression. Moreover, the size of the dot represents the number of cells expressing this kind of gene. **(L**–**N)** AKR1B10 induces therapeutic resistance in HCC cells (L), and colony formation assays (M) were used to assess the role of AKR1B10 shRNA and fidarestat on the sensitivities of HepG2Sora or HepG2OXA cells to sorafenib and oxaliplatin. (N) CCK-8 assay was used to assess the role of AKR1B10 shRNA and fidarestat on the sensitivities of Huh-7Sora or Huh-7OXA cells to sorafenib and oxaliplatin. All data were expressed as mean ± standard deviation of three independent experiments. ∗*P* < 0.05, ∗∗*P* < 0.01, and ∗∗∗*P* < 0.001.

Secondly, a six-gene prognostic signature for HCC therapy resistance was developed and validated. LASSO and stepwise regression of 15 hub genes identified six biomarkers (Fig. 1C; Fig. S4A and S4B). The risk score formula integrated weighted expression: Risk score = (0.19 × *LOX*) + (0.233 × *AKR1B10*) + (0.156 × *ALPP*) + (−0.142 × *CFB*) + (0.207 × *NDRG1*) + (0.143 × *ODC1*). Using median scores, TCGA-LIHC (training), GSE14520, and ICGC-LIRI-JP (validation) cohorts were stratified into high-risk/low-risk groups. High-risk patients had significantly shorter overall survival (*P* < 0.0001; Fig. 1D–F; Fig. S4C and S4D). The model demonstrated robust accuracy, with the values for the area under the curve greater than 0.7 for 1-, 3-, and 5-year survival (Fig. 1D–F; Fig. S4C and S4D). *AKR1B10*, *LOX*, *ALPP*, *NDRG1*, and *ODC1* were risk factors; *CFB* was protective (Fig. 1D–F; Fig. S4C and S4D). Compared with three existing models, the six-gene signature demonstrated superior performance (higher C-index; Fig. S4E), underscoring enhanced clinical utility for stratifying sorafenib/oxaliplatin-resistant HCC patients. Its prognostic power stems from direct linkage to resistance mechanisms, enabling tailored risk assessment and guiding therapy.

Thirdly, we identified the independent clinical value of the as-constructed model incorporating six genes related to oxaliplatin/sorafenib resistance. The risk score and the clinical data of the training cohort were subjected to univariate and multivariate Cox regression analyses. Samples in the training cohort were subjected to univariate analysis (Fig. 1G). Patients with stage III/IV tumors exhibiting high risk scores were associated with poor prognosis. Multivariate analysis revealed that the only risk scores (Fig. 1H; hazard ratio = 2.27, 95% CI = 1.51–3.40, *P* < 0.001) and clinical stage (hazard ratio = 2.21, 95% CI = 1.50–3.26, *P* < 0.001) were independent predictive factors of prognosis. These findings suggest that the six-gene signature can independently predict HCC prognosis.

Fourthly, functional enrichment in HCC risk groups was analyzed. Single-sample Gene Set Enrichment Analysis revealed six pathways differentially enriched: fatty acid metabolism, primary bile acid biosynthesis, and peroxisome were inversely correlated with risk scores; cell cycle and *E. coli* infection were positively correlated (Fig. S5A), implicating proliferation, metabolic reprogramming, and redox imbalance in resistance and prognosis. The correlation between the six-gene signature and cellular oxidative stress regulation was examined. Using single-sample Gene Set Enrichment Analysis, multiple oxidative stress pathways in TCGA-LIHC were negatively correlated with tumor grade/age (Fig. S5B). The activation differed between risk groups and resistance-related subtypes: oxidative stress pathways were down-regulated in the favorable-prognosis C2 subtype and low-risk group (Fig. S5C and S5D). Genes *AKR1B10*, *LOX*, and *ODC1* were significantly positively correlated with multiple oxidative stress pathways (Fig. S5E). This partially confirms oxidative stress regulation's close association with HCC therapy resistance/prognosis, warranting further study of these genes' regulatory roles.

Fifthly, we verified the expression of the six prognostic genes by immunohistochemical staining (Table S1) and single-cell RNA-sequencing dataset GSE242889. Immunohistochemistry analysis revealed elevated tumor levels of *AKR1B10*, *LOX*, *NDRG1*, *ALPP*, and *ODC1*, while *CFB* was down-regulated, with AKR1B10 showing the largest tumor-to-normal disparity (Fig. 1I; Fig. S6A). Single-cell RNA-sequencing analysis highlighted *AKR1B10*, *NDRG1*, and *ALPP* enrichment in malignant cells (Fig. 1J and K; Fig. S6B). These findings were consistent with the RNA sequencing analysis of the expression of six therapy resistance-related prognostic genes in the TCGA dataset.

Finally, we examined AKR1B10's role in conferring HCC therapy resistance. Quantitative reverse transcription PCR analysis (Table S2) of six signature genes in HepG2 versus HepG2^Sora^/HepG2^OXA^ cells revealed that only AKR1B10 was significantly up-regulated in both resistant lines (Fig. S7A). Targeting AKR1B10 via shRNA knockdown or fidarestat (activity inhibitor) significantly increased resistant cell sensitivity to sorafenib and oxaliplatin (HepG2^Sora^/HepG2^OXA^: Fig. S7B and Fig. 1L and M; Huh-7^Sora^/Huh-7^OXA^: Fig. 1N). These results demonstrate that AKR1B10 promotes sorafenib and oxaliplatin resistance in HCC cells.

This study established a six-gene signature associated with oxaliplatin and sorafenib resistance in HCC, constructing a prognostic risk model. The validations employing single-cell RNA sequencing (GSE242889) and immunohistochemistry confirmed AKR1B10 as a critical driver of therapeutic resistance. The signature effectively stratifies HCC patients into subgroups exhibiting divergent prognostic outcomes, thereby facilitating personalized therapeutic strategies: elevated risk scores may warrant alternative neoadjuvant regimens, whereas favorable profiles could proceed directly to systemic therapies. This signature functions as a robust biomarker for prognostication and treatment personalization in therapy-resistant HCC. Furthermore, the model elucidates underlying resistance mechanisms, including dysregulated drug metabolism and oxidative stress pathways, informing the rational design of targeted inhibitors or synergistic combination therapies. These findings provide a bioinformatics foundation with significant clinical implications for overcoming therapeutic resistance.

This project innovatively develops a pioneering HCC resistance-gene prognostic model. It surpasses existing multi-gene models via precise individualized drug guidance while uniquely exploring resistance mechanisms. Our analysis confirms that the signature, especially AKR1B10, robustly correlates with treatment resistance and prognosis, aligning with prior evidence. However, current knowledge lacks a detailed understanding of these genes' intricate pathways. Future research should investigate specific molecular mechanisms driving drug resistance. Additionally, exploring targeted therapies to overcome/reverse resistance warrants investigation. Integrating this signature into clinical practice and its treatment impact requires validation through large-scale trials.

## CRediT authorship contribution statement

**Siliang wang:** Writing – original draft, Funding acquisition, Conceptualization. **Shuangshuang Wang:** Writing – original draft, Methodology. **Xu Wang:** Methodology, Formal analysis. **Yuxiang Sun:** Resources, Data curation. **Xiao Du:** Visualization. **Dan Han:** Validation. **Huanyu Ni:** Writing – review & editing. **Yun Zhu:** Resources, Funding acquisition. **Huilian Shi:** Writing – review & editing, Supervision. **Zhaocong Yang:** Writing – review & editing, Visualization.

## Ethics declaration

The collection and use of human samples were approved by the Ethics Committee of Affiliated Hospital of Nanjing University of Chinese Medicine (No. 2021NL-099-01) following the Declaration of Helsinki ethical guidelines.

## Fundings

This study was financially supported by the 10.13039/501100001809National Natural Science Foundation of China (No. 82374223), and the Jiangsu Provincial Double-Innovation Doctor Program (China) (No. PF-302-2021).

## Conflict of interests

The authors declared no conflict of interests.
