# Supplementary material for: Identification of a novel prognostic gene signature associated with therapeutic resistance in hepatocellular carcinoma
Source: Genes Dis. 2025 Aug 30;13(4):101837. doi: 10.1016/j.gendis.2025.101837 (PMC13011024; doi:10.1016/j.gendis.2025.101837)
Supplement: Multimedia component 1 [file mmc1.docx]

**Supporting Information**

**Availability of data and material**

The dataset used in this study is available in GSE62813 (https://www.ncbi.nlm.nih.gov/geo/query/acc.cgi?acc=GSE62813), GSE129071 (https://www.ncbi.nlm.nih.gov/geo/query/acc.cgi?acc=GSE129071), TCGA, GSE14520 (https://www.ncbi.nlm.nih.gov/geo/query/acc.cgi?acc=GSE14520) and ICGC-LIRI-JP (https://dcc.icgc.org/projects/LIRI-JP).

**Materials and methods**

***Data collection and study design***

The gene expression profiles of HCC cells with differential drug sensitivities were acquired from the GSE62813 (sorafenib) and GSE129071 (oxaliplatin) datasets. The scan data was imported using GeneSpring GX and subjected to quartile normalization.

To develop a prognostic model, patients meeting the following inclusion criteria, in accordance with the REMARK guidelines, were considered: (a) a histologically confirmed diagnosis of hepatocellular carcinoma (HCC); (b) availability of overall survival (OS) data and RNA sequencing data; and (c) data that could be accessed and obtained from our institution or public databases. This study obtained three datasets (The Cancer Genome Atlas-Liver Hepatocellular Carcinoma (TCGA-LIHC), GSE14520, and International Cancer Genome Consortium (ICGC)-LIRI-JP) comprising the data of clinical features, gene expression, and prognosis. The TCGA-LIHC (n=374) dataset was utilized as the training cohort, while the ICGC-LIRI-JP (n=231) and GSE14520 (n=221) datasets functioned as validation cohorts. This study also obtained 17 fresh HCC tissues from our own institution (approved by the Ethics Committee of Affiliated Hospital of Nanjing University of Chinese Medicine (NO.2021NL-099-01) following the Declaration of Helsinki ethical guidelines) (Table S1).

***Analysis of differentially expressed genes (DEGs) and functional annotations***

DEGs between sorafenib/oxaliplatin-sensitive and sorafenib/oxaliplatin-resistant HCC cells were identified using the limma package based on the following criteria: |log2 fold change (FC)| > 1, false discovery rate (FDR) < 0.05, and P < 0.05. Additionally, the heatmap and volcano plot were plotted using pheatmap and limma packages, respectively. Functional enrichment analysis of DEGs was performed using the clusterProfiler package based on the following criteria: FDR < 0.05 and P < 0.05.

***HCC subtype identification based on genes related to therapy resistance***

Consensus clustering analysis was performed using the ConsensusClusterPlus package. Molecular subtypes were selected based on sorafenib/oxaliplatin resistance-related genes. Euclidean distance (ED) was determined for analyzing the similarity distance between different samples. K-means was used to repeat cluster analysis 500 times, and 80% of samples were used in each repeat. The cumulative distribution function curves were generated and the consensus scores were calculated to determine the optimal cluster number. Two subtypes were compared using SigClust analysis to determine the clustering significance.

***Therapy resistance-related hub gene selection and risk assessment model construction***

The online tool Search Tool for the Retrieval of Interacting Genes (STRING) was used to construct a protein-protein interaction (PPI) network with therapy resistance-related genes associated with prognoses. The central nodes (hub genes) in the network were determined by calculating various topological parameters. The candidate genes were selected for model construction using least absolute shrinkage and selection operator (LASSO) Cox regression with the glmnet and survival packages. The glmnet package determines the penalty parameter lambda using cross-validation. The optimal lambda related to the least cross-validation error mean was determined. Next, the best gene group (lambda = 0.0236) was selected to construct the model. The stepAIC approach from the MASS package was adopted to perform stepwise multivariate regression using gene expression data. The gene sets with the most suitable Akaike Information Criterion values were selected for constructing the HCC prognosis prediction model. The LASSO Cox regression coefficients were combined linearly to calculate the risk scores based on corresponding gene levels. Patients were classified into high-risk and low-risk groups. The receiver operating characteristic (ROC) curve involving clinical features was plotted to assess the specificity and sensitivity of the risk model and calculate the area under the curve (AUC) values.

***Gene set enrichment analysis (GSEA)***

The correlation of the risk score with signaling pathways, drug response, and cellular oxidative stress was examined. The expression data of relevant genes in samples from the training cohort were used to perform single-sample GSEA with the R package GSVA function. The scores of diverse samples in different drugs and pathways were calculated. In particular, ssGSEA scores for all samples in regulatory drugs and pathways were determined. This study also analyzed the relationship between these scores and risk scores based on the following criteria: FDR < 0.5.

***Clinical sample preparation and immunohistochemical analysis***

The tissues were fixed in 10% formalin at room temperature, dehydrated, paraffin-embedded, and sectioned to a thickness of 4 μm. The sections were deparaffinized in xylene, dehydrated in an alcohol series, blocked with 20% horse serum, and incubated with the primary antibodies at 4 °C overnight. After washing with phosphate-buffered saline (PBS), the sections were incubated with a biotinylated universal secondary antibody. Next, the sections were washed and incubated with the avidin-biotin detection complex. Immunoreactive signals were developed using a 3,3′-diaminobenzidine solution. Immunohistochemical staining was semiquantitatively analyzed using Image J. The percentage of positive cells was scored on a scale of 1–6 as follows: score 1, 0%–10% of positive cells; score 2, 11%–30% of positive cells; score 3, 31%–50% of positive cells; score 4, 51%–70% of positive cells; score 5, 71%–90% of positive cells; score 6, 91%–100% positive cells.

***Single-cell RNA transcriptome data analysis***

We downloaded scRNA-seq dataset GSE242889 from GEO database and obtained 46,789 cells from 5 pairs of HCC and adjacent non-tumor tissues. For cell type identification, we collected ineage-specific markers to categorize main celltypes (CD3E for T cells, KLRD1 for NK cells, CD79A for B cells, LYZ for myeloid-cells,PECAM1 for endothelial cells,ACTA2 for mesenchymal-cells, Transferrin for malignant cellsand EPC,AM for hepatic progenitor cells). We utilized the RunHarmony to remove batch effects across different patients for immune and stromal cells, but not for malignant and hepatic progenitor cells.

***Cell culture***

The HCC cell lines used in this study were HepG2 (purchased from the American Type Culture Collection) and Huh-7 (purchased from the Shanghai Cell Bank, Chinese Academy of Sciences) cells. Sorafenib-resistant (HepG2^Sora^ and Huh-7^Sora^) or oxaliplatin-resistant (HepG2^OXA^ and Huh-7^OXA^) cell lines were generated by continuously treating HepG2 and Huh-7 cells with sorafenib or oxaliplatin (up to 10 μM), respectively, to mimic the clinical phenomenon of acquired resistance.

***Reagents***

The reagents used in this study were sorafenib (Bayer-Schering Pharma), oxaliplatin (MedchemExpress), and fidarestat (AKR1B10 inhibitor, MedchemExpress). Short hairpin RNA (shRNA) targeting *AKR1B10* or shRNA control (pLKO.1-puro-CMV-tGFP vector, Sigma) was transfected using Lipofectamine 3000 reagent (Invitrogen), following the manufacturer’s instructions.

***Cell viability assay***

Cells cultured in Dulbecco’s modified Eagle’s medium (DMEM) containing dimethyl sulfoxide (0.01%) were used as negative controls. The medium served as the blank control. The drug-resistant and drug-sensitive cell lines (100 μL, approximately 5000 cells) were seeded into 96-well plates containing the indicated concentrations of drugs and cultured at 37 °C and 5% CO_2_ for 24 h. Next, the cells were incubated with 10 μL of cell counting kit-8 (Sigma) solution for 4 h. The absorbance at 450 nm of the reaction mixture was measured using a microplate reader.

***Western blot analysis.***

Whole-cell lysates were prepared with RIPA lysis buffer containing protease and phosphatase inhibitors. Cell lysates were loaded on SDS-PAGE and transferred onto PVDF membranes. After the membranes were blocked with 5% skimmed milk at room temperature for 2–3 hours, they were incubated with antibody against AKR1B10 (1: 1000, Abcam) and GAPDH (1: 6000, Bioworld Technology), followed by incubation with Goat anti-Rabbit IgGs-HRP (1: 10000, Bioworld Biotechnology). The target proteins were detected using the ECL system (Millipore) and visualized using the ChemiDoc XRS system (Bio-Rad).

***Colony formation assay***

Colony formation was examined using a plate colony formation assay. Cells at the logarithmic phase were seeded in duplicate at a density of 800 cells/well in flat-bottom plates containing 3 mL DMEM supplemented with 10% fetal bovine serum and treated with or without drugs for 14 days at 37 °C and 5% CO_2_. The colonies were fixed in 100% ice-cold ethanol and subjected to crystal violet staining. The number of colonies (>50 cells) was counted, and the average number of colonies from three independent experiments (with duplicate wells per condition) was represented.

***Quantitative real-time polymerase chain reaction (qRT-PCR) analysis***

Total RNA was extracted using Trizol (Invitrogen) and reverse-transcribed into complementary DNA using the Super Script First-Strand Synthesis System (Roche). qRT-PCR analysis was performed using Roche 480 with SYBR Green I Master (Roche). The primer sequences used in this study are listed in Table S2. *GAPDH* served as an internal reference. The relative expression of target genes was calculated using the 2^–ΔΔCt^ method.

***Statistical analysis***

Statistical analyses were performed using R software. Data are represented as median values. The OS of the low-risk and high-risk groups was analyzed using the Kaplan-Meier (KM) method and compared using the log-rank test. The Cox proportional hazard regression model was used to perform univariate and multivariate analyses. Datasets were merged with the merge script in Perl language. Differences were considered significant at P < 0.05.

**Supplementary materials**

**Table S1.** Clinical information of 17 HCC samples.

**Table S2.** Gene primers applied in qRT-PCR analysis.

**Table S3.** DEGs between HCC cells with sorafenib resistance and sensitivity.

**Table S4.** DEGs between HCC cells with oxaliplatin resistance and sensitivity.

**Table S5.** Identification of 27 key prognostic genes related to therapeutic resistance.


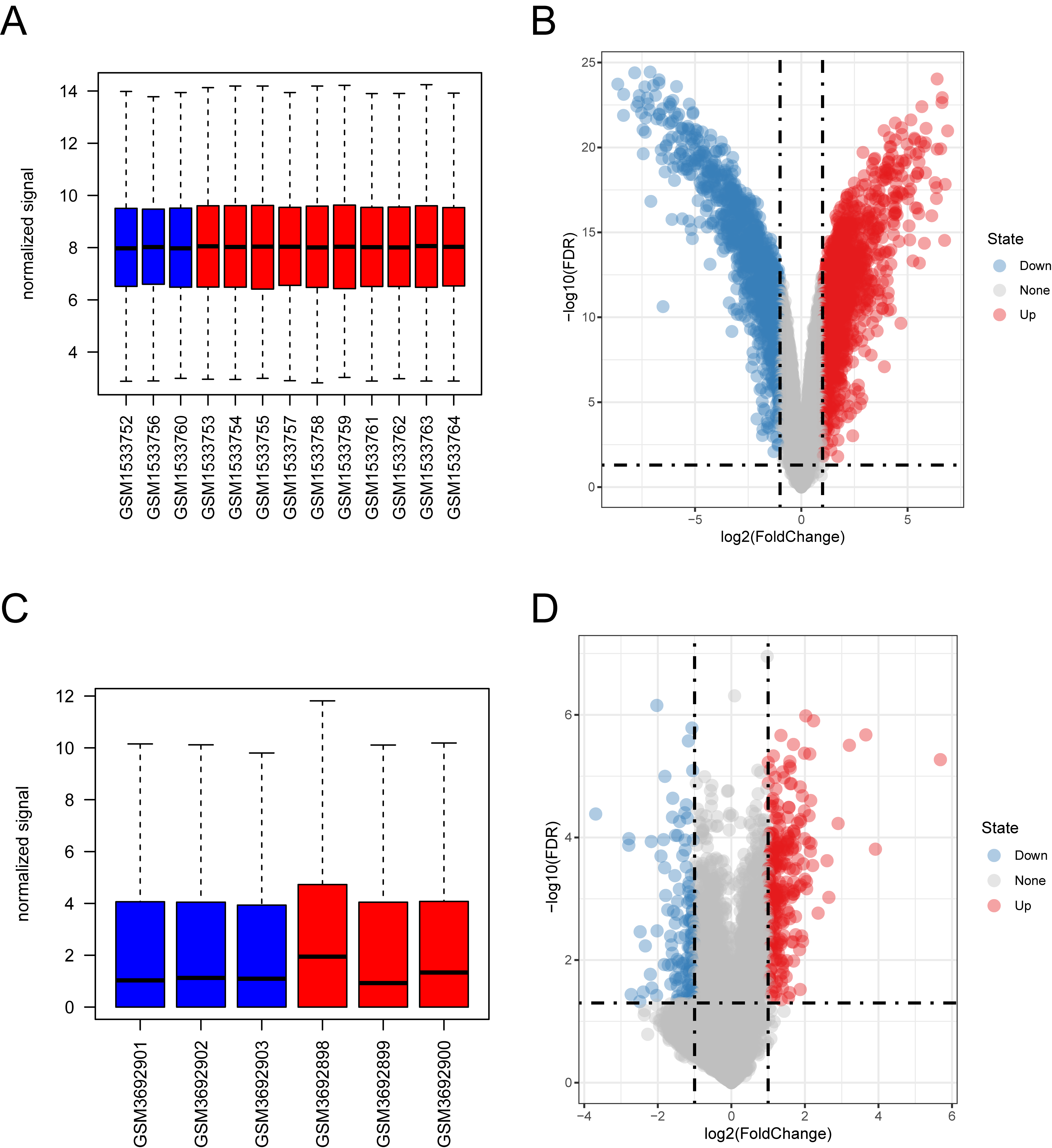


**Fig.S1 DEGs between HCC cells with sorafenib/oxaliplatin resistance and sensitivity.** (A) Box plot showing the distributions of expression data in sorafenib resistant and sensitive HCC cells. (B) Volcano gram showing DEGs between HCC cells with sorafenib resistance and sensitivity. (C) Box plot showing the distributions of expression data in oxaliplatin resistant and sensitive HCC cells. (D) Volcano gram showing DEGs between HCC cells with oxaliplatin resistance and sensitivity.


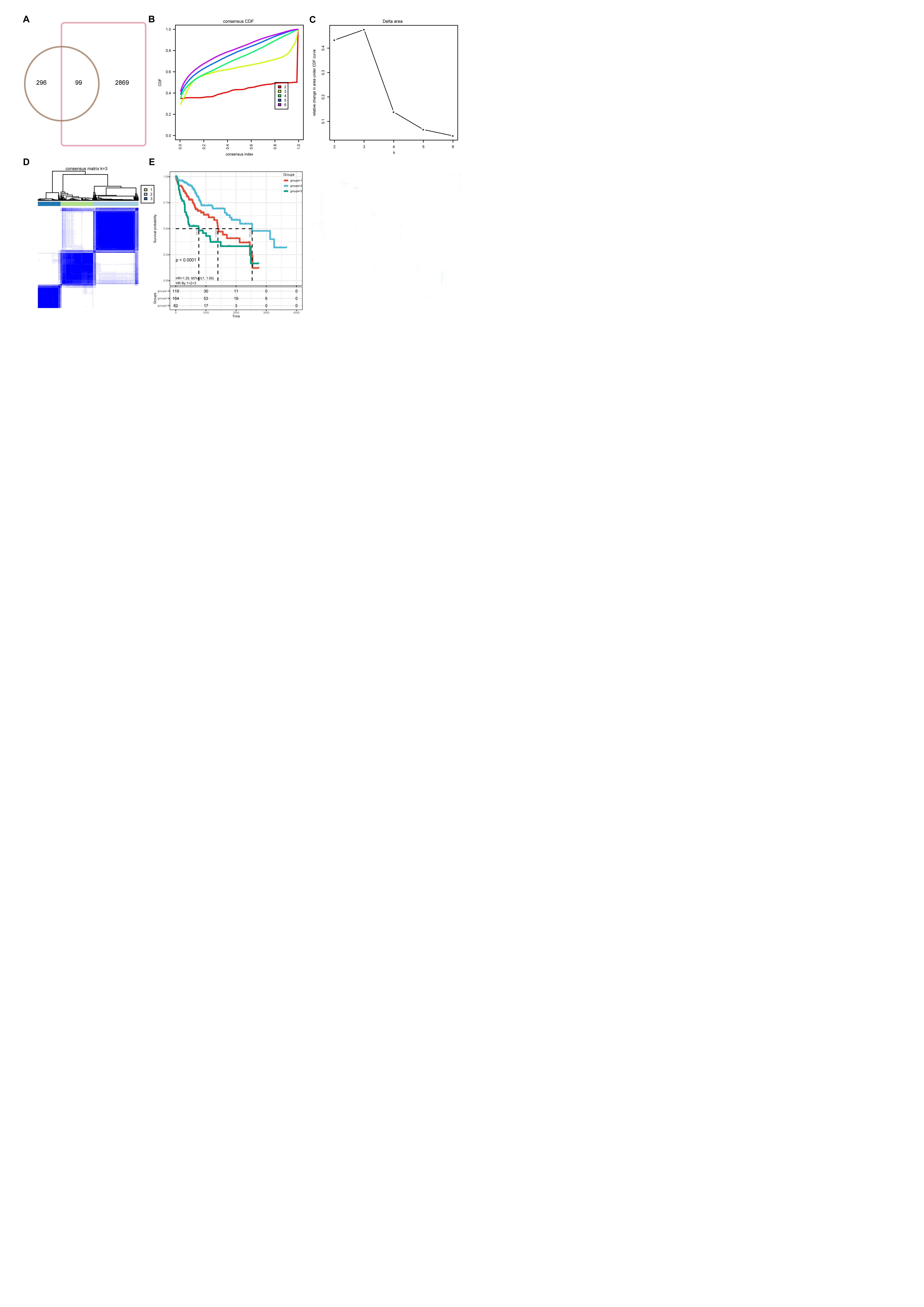


**Fig.S2 HCC samples in training set classified based on genes related to treatment resistance through consistent cluster analysis.** (A) Venn diagram shows the 99 genes related to both oxaliplatin and sorafenib resistance. (B) CDF curves showing the consensus scores according to diverse numbers of subtypes (k=2~6), and respective colors are shown to facilitate deciding the optimal k for obtaining the maximal CDF (in other words, to obtain the optimal cluster confidence and consistency). (C) Comparisons of relative changes in AUC values of CDF curve between k and k−1, thus assisting in determining relative increases in consensus and significantly increased k value. (D) Consensus score matrix for HCC samples at k = 3 (1 = C1, 2 = C2, 3 = C3). (E) Difference in prognosis of samples classified based on consistent cluster analysis in the training set.


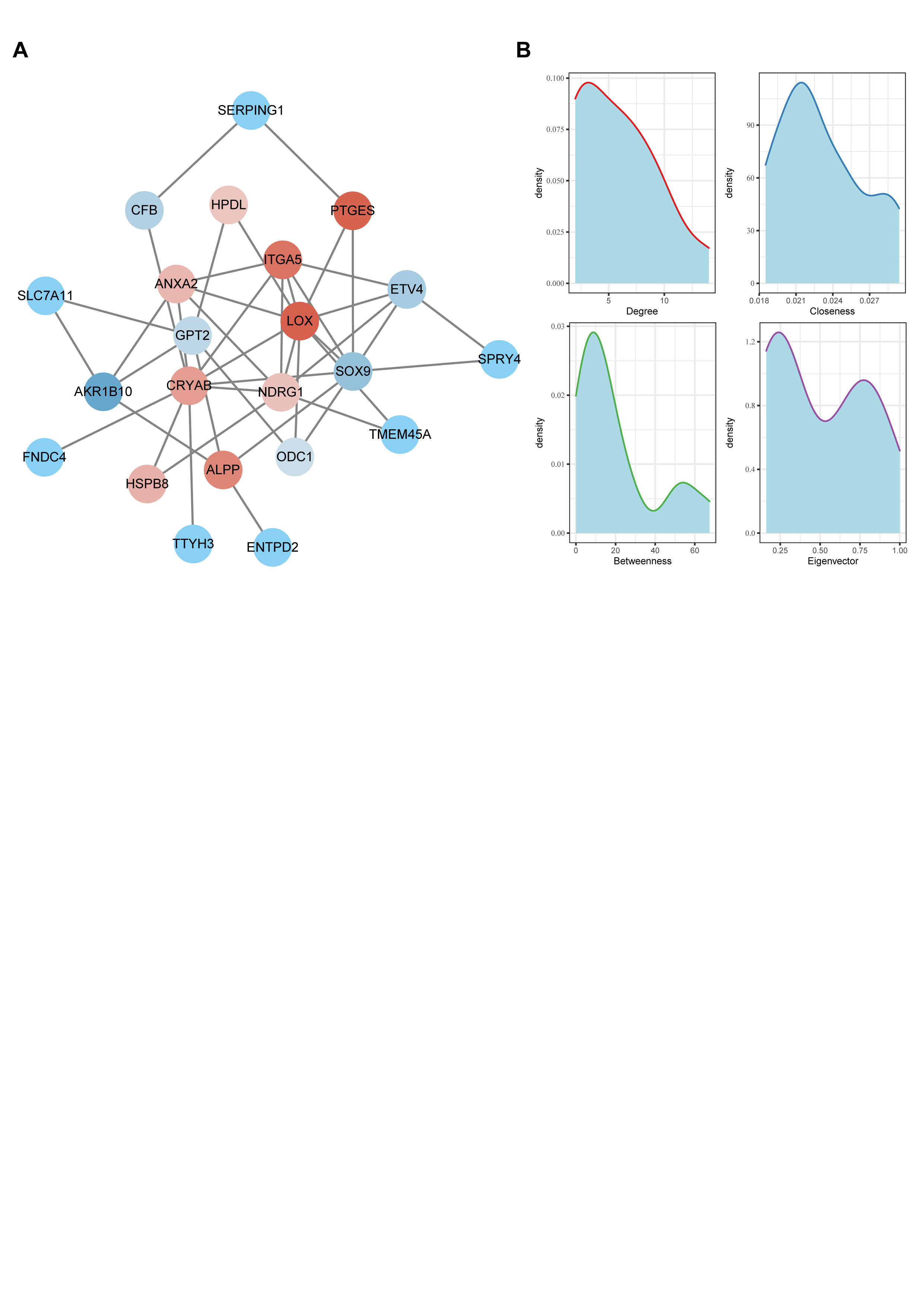


**Fig.S3 Determination of critical prognosis-related DEGs between treatment-sensitive and treatment-resistant HCC cells.** (A) PPI network constructed based on therapeutic resistance-related prognostic genes. (B) The distribution of degree, closeness, betweenness and eigenvector centrality of genes within the network.


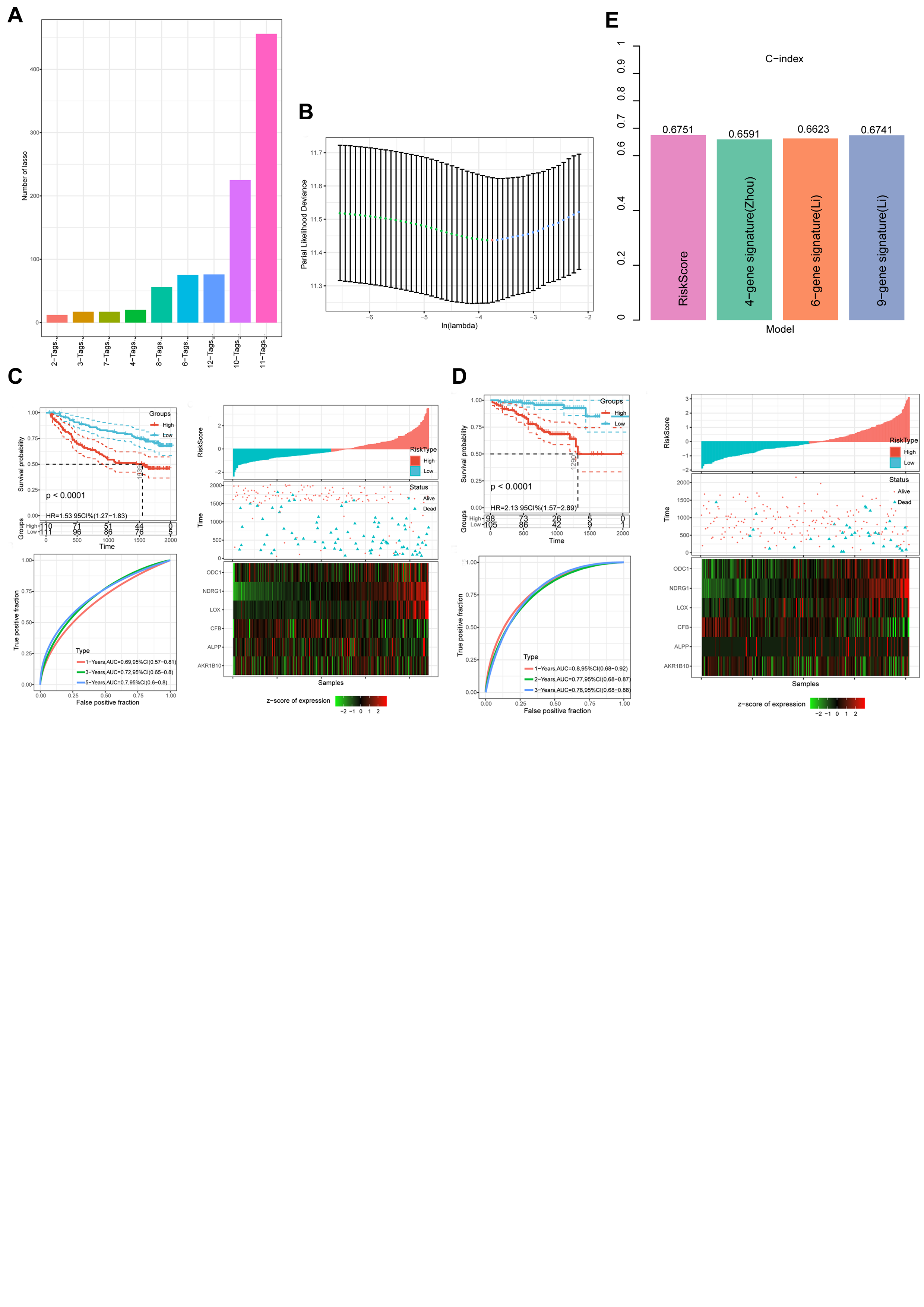


**Fig.S4 Validation of the prognosis prediction model constructed based on genes related to treatment resistance.** (A) Gene combination frequencies upon 1000 iterations of LASSO regression. (B) CIs for every lambda. Lambda that had the lowest MSD was selected to be the optimal model; in other words, lambda=0.0236. (C-D) Performance of our as-constructed prognosis model based on 6 therapeutic resistance-related genes in classifying test set samples (C: GSE14520 and D: ICGC-LIRI-JP): Difference in prognosis after classification of HCC samples using our 6-gene signature. ROC curves for the 6-gene signature for HCC sample. Associations between risk score, survival status, survival time and 6 gene expression levels within HCC samples. (E) Comparison of the treatment resistance-related risk model with other known HCC prognosis prediction models reported in literature. C-index (concordance index) was used to evaluate the predictive ability of the 4 models.


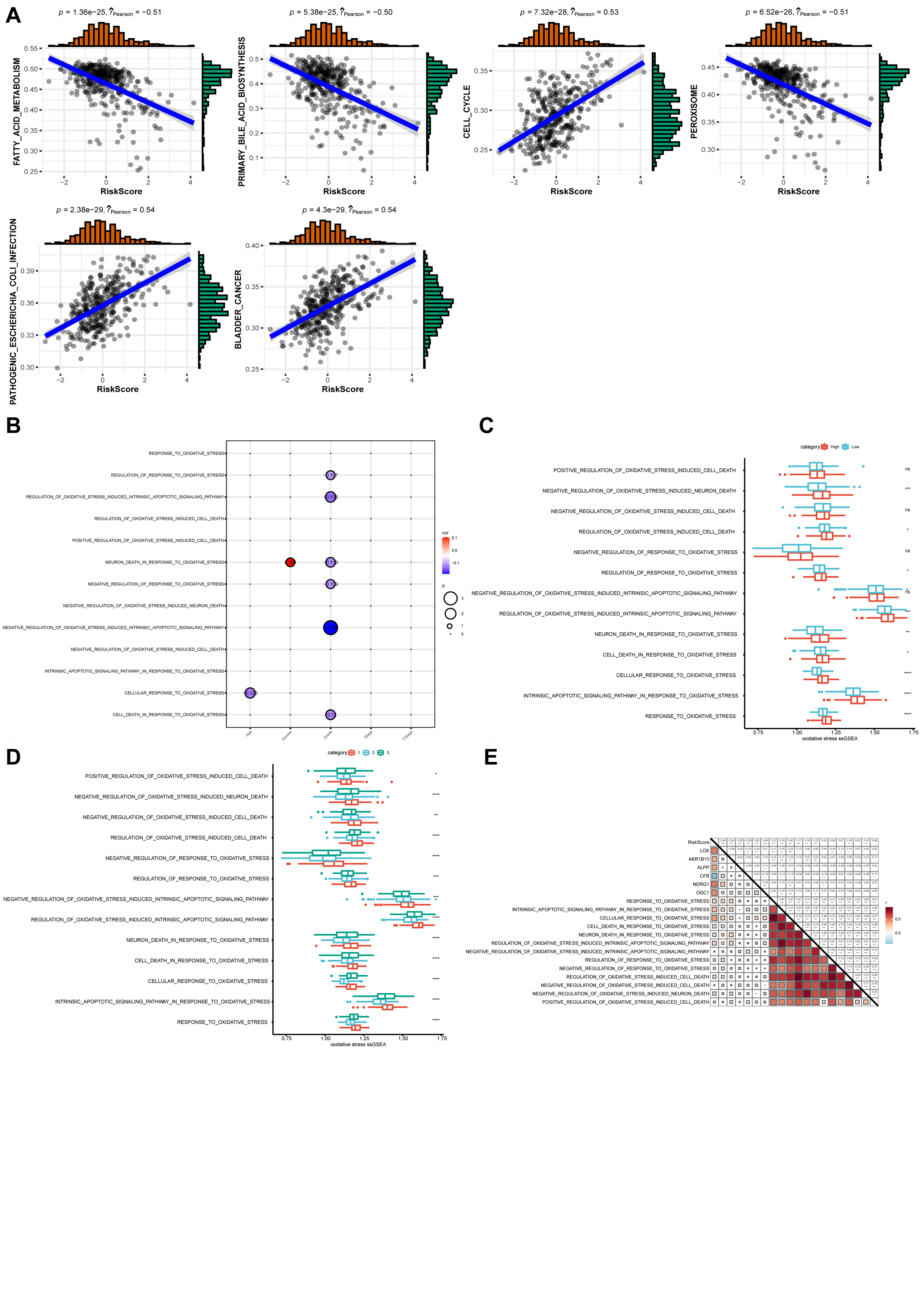


**Fig.S5 ssGSEA of differential pathways between the high-risk and low-risk groups.** (A) Differences in those enriched pathways between low- and high-risk groups. (B-E) Relation between the ability of cells to regulate oxidative stress and constructed risk model: (B) Correlation between the levels of oxidative stress pathways and clinicopathological features in TCGA-LICH cohort. (C) Differences in levels of oxidative stress pathways between samples with divers risk scores. (D) Differences in levels of oxidative stress pathways between samples in different treatment resistant subtypes. (E) Correlation between the levels of risk scores, LOX, AKR1B10, ALPP, NDRG1, ODC1, CFB and oxidative stress pathways.


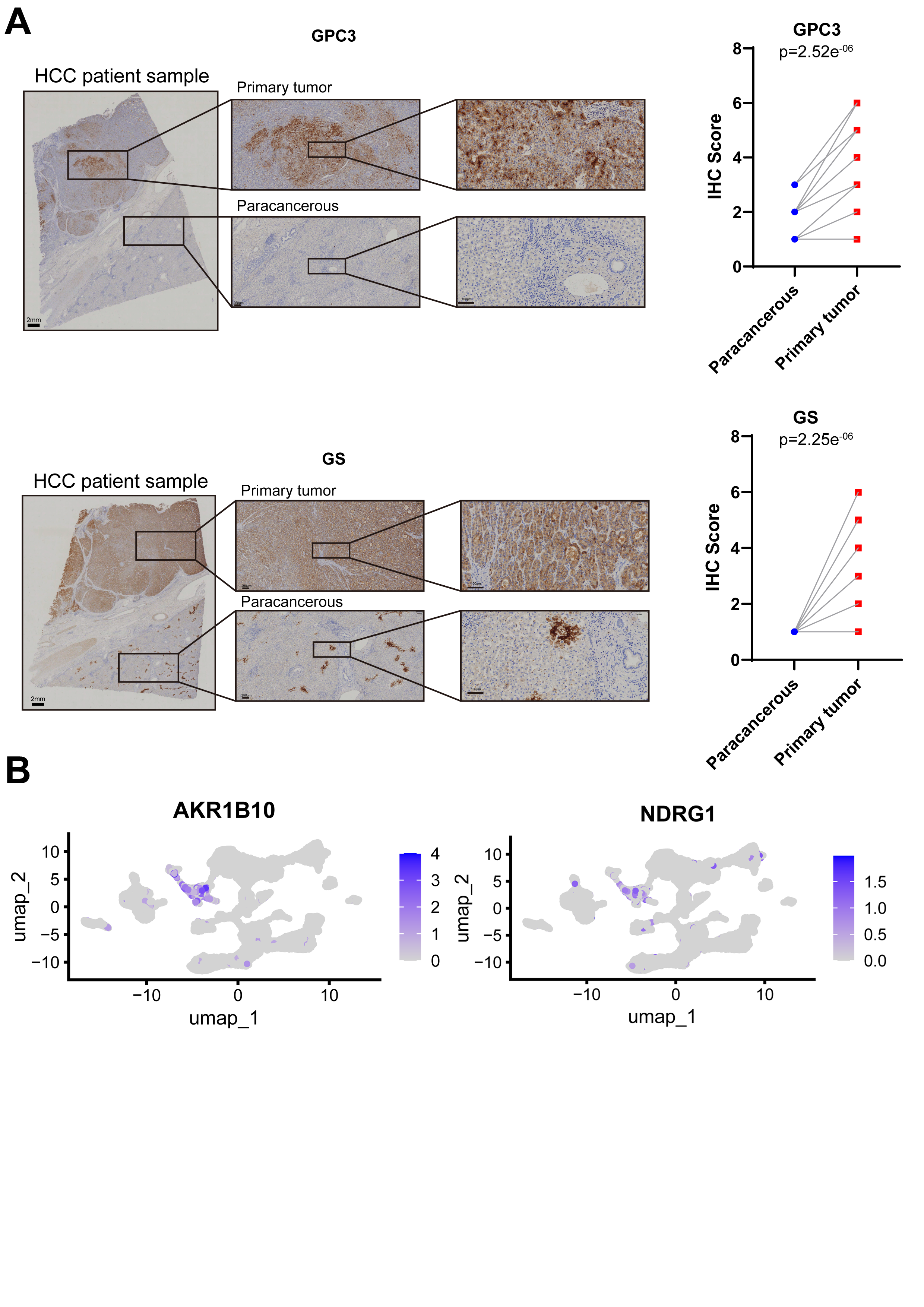


**Fig.S6 Verification the expression of the six prognostic genes by immunohistochemical staining and scRNA-seq dataset GSE242889.** (A) Levels of GS and GPC3 in paracancerous and primary HCC tumor samples detected by IHC. Right, IHC scores of the proteins. (B) UMAP showed the expression of AKR1B10 and NDRG1.


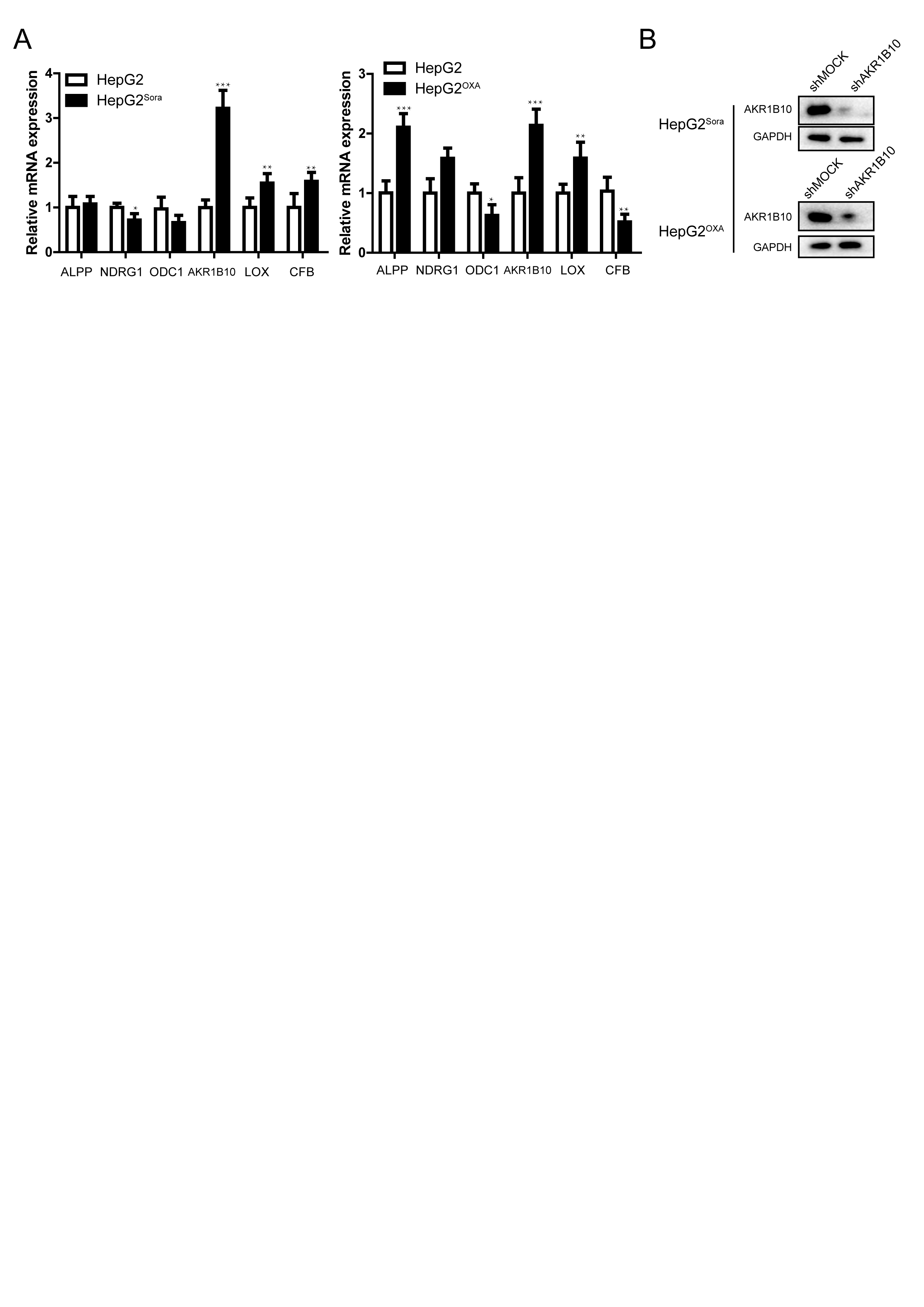


**Fig.S7** (A) The differential expression levels of these genes between HepG2 cells and HepG2^Sora^/HepG2^OXA^ cells by qRT-PCR assay. (B) The interference efficiency of shRNA was verified by WB. All data are expressed as mean ± SD of three independent experiments; * P < 0.05, ** P < 0.01, *** P < 0.001.
